# High Plasma Levels of Activated Factor VII-Antithrombin Complex Point to Increased Tissue Factor Expression in Patients with SARS-CoV-2 Pneumonia: A Potential Link with COVID-19 Prothrombotic Diathesis

**DOI:** 10.3390/diagnostics12112792

**Published:** 2022-11-14

**Authors:** Nicola Martinelli, Anna Maria Rigoni, Sergio De Marchi, Nicola Osti, Martino Donini, Martina Montagnana, Annalisa Castagna, Patrizia Pattini, Silvia Udali, Lucia De Franceschi, Elisa Tinazzi, Filippo Mazzi, Sara Moruzzi, Giuseppe Argentino, Lorenzo Delfino, Giulia Sartori, Anna Maria Azzini, Evelina Tacconelli, Patrick Van Dreden, Giuseppe Lippi, Domenico Girelli, Oliviero Olivieri, Simonetta Friso, Francesca Pizzolo

**Affiliations:** 1 Department of Medicine, University of Verona, 37129 Verona, Italy; 2 Angiology Unit, Department of Cardiovascular and Thoracic, Azienda Ospedaliera Universitaria Integrata, 37126 Verona, Italy; 3 Section of Clinical Biochemistry, University of Verona, 37129 Verona, Italy; 4 Department of Diagnostics and Public Health, University of Verona, 37129 Verona, Italy; 5 Clinical Research Department, Diagnostica Stago, 92230 Gennevilliers, France

**Keywords:** severe acute respiratory syndrome coronavirus 2 (SARS-CoV-2), coronavirus disease 2019 (COVID-19), coagulation, tissue factor (TF), activated factor VII-antithrombin (FVIIa-AT)

## Abstract

Severe acute respiratory syndrome coronavirus 2 (SARS-CoV-2) is the causal agent of coronavirus disease 2019 (COVID-19), in which coagulation abnormalities and endothelial dysfunction play a key pathogenic role. Tissue factor (TF) expression is triggered by endothelial dysfunction. Activated factor VII-antithrombin (FVIIa-AT) complex reflects indirectly FVIIa-TF interaction and has been proposed as a potential biomarker of prothrombotic diathesis. FVIIa-AT plasma concentration was measured in 40 patients (30 males and 10 females; 64.8 ± 12.3 years) admitted with SARS-CoV-2 pneumonia during the first pandemic wave in Italy. Two sex- and age-matched cohorts without COVID-19, with or without signs of systemic inflammation, were used to compare FVIIa-AT data. The FVIIa-AT plasma levels in COVID-19 patients were higher than those in non-COVID-19 subjects, either with or without inflammation, while no difference was observed among non-COVID-19 subjects. The association between COVID-19 and FVIIa-AT levels remained significant after adjustment for sex, age, C-reactive protein, renal function, fibrinogen, prothrombin time and activated partial thromboplastin time. Our results indicate that SARS-CoV-2 infection, at least during the first pandemic wave, was characterized by high FVIIa-AT levels, which may suggest an enhanced FVIIa-TF interaction in COVID-19, potentially consistent with SARS-CoV-2-induced endotheliopathy.

## 1. Introduction

Severe acute respiratory syndrome coronavirus 2 (SARS-CoV-2) is an enveloped, single-stranded RNA virus. Highly transmissible and pathogenic in humans, this virus originated the pandemic of coronavirus disease 2019 (COVID-19), which has revolutionized world perspectives during the past two years, affecting over 600 million subjects and causing nearly 6.5 million worldwide deaths so far [1]. Although originally characterized as a primarily respiratory illness, COVID-19 is not limited to the respiratory tract and has progressively been revealed as a multiorgan and multifaceted disease, encompassing several different clinical manifestations, from a totally asymptomatic or mild flu-like syndrome to interstitial pneumonia, up to severe multiorgan failure [2,3,4]. 

In this rather heterogeneous clinical context, alterations in the coagulation pathway and thrombotic risk have a prominent place. During the first pandemic wave, several studies showed a disproportionate higher prevalence of abnormal coagulation tests and thrombotic events in both critically ill and non-critically ill COVID-19 patients. High rates of venous thromboembolism and arterial thrombosis (up to 30%) were reported [5,6,7,8,9,10]. Concomitantly, several studies reported important laboratory abnormalities including increased levels of D-dimer, fibrin degradation products (FDP), fibrinogen, and von Willebrand factor (VWF), as well as prolonged prothrombin time (PT) and activated partial thromboplastin time (aPTT) [11,12,13]. Some of these abnormalities were considered consistent with a condition of diffuse intravascular coagulation (DIC) in the setting of SARS-CoV-2-induced inflammatory response. However, unlike patients with DIC, in patients with COVID-19 fibrinogen plasma levels are generally normal and even often increased, clinically apparent bleeding episodes are very infrequent, and classic DIC has been reported in only a few patients, especially in those with advanced/terminal stages of disease [11]. Reliable evidence thus suggests that COVID-19 patients may be characterized by marked hypercoagulability rather than consumption coagulopathy. Thromboelastometry profiles in COVID-19 patients were in keeping with this assumption [14]. Antiphospholipid antibodies have also been detected in COVID-19 patients [15]. On the other hand, the VWF/ADAMTS13 imbalance, with elevated levels of VWF and decreased ADAMTS13 activity [13,16] and complement-induced coagulopathy [17] recalls the characteristics of thrombotic microangiopathy and, beyond macrovascular thrombosis, microvascular thrombosis is a prominent feature in the pathophysiology of both lung and multi-organ complications triggered by SARS-CoV-2 infection [17,18]. 

Endothelial dysfunction, which plays a key role in COVID-19, is a pathologic condition coupling hypercoagulability and inflammatory status [19]. An endotheliopathy-related pathophysiology of COVID-19 appears plausible for many biological reasons. SARS-CoV-2 can directly infect vascular endothelial cells, thereby leading to either cellular damage/apoptosis or inflammatory alterations [20,21,22]. Endothelial cells may be activated by cytokines in the pro-inflammatory storm seen in severe forms of SARS-CoV-2 infection, thereby decreasing the antithrombotic activity of normal endothelium and fostering procoagulant characteristics [17,19]. Furthermore, according with the interplay between inflammation and coagulation, the COVID-19-related cytokine storm has been shown to modulate the activity of proteases and cofactors of the coagulation cascade [23,24]. In the complex pathways of immunothrombosis, the activation of platelets and neutrophils may lead to enhanced neutrophil extracellular trap (NET) generation, while hypoxia-inducible transcription factors may upregulate tissue factor (TF) expression, further contributing to COVID-19-associated prothrombotic diathesis [19]. Activated factor VII-antithrombin (FVIIa-AT) complex is generally considered a reliable indirect marker of TF-FVIIa interaction, which has been associated with thrombophilic diathesis and an increased risk of mortality in cardiovascular cohorts [25,26,27]. 

Bearing in mind that (i) TF expression is characteristic in endothelial dysfunction and inflammatory damage [28] and (ii) COVID-19 is characterized by endotheliopathy [17,19], we hypothesized that FVIIa-AT plasma levels may be increased in patients with SARS-CoV-2 infection. To test this working hypothesis, we assessed FVIIa-AT plasma concentration in 40 consecutive patients with SARS-CoV-2 pneumonia admitted to COVID-19 Standard Care Units during the first pandemic wave in April 2020. Laboratory data were then compared with those of two historical control groups, the former composed by subjects comparable for sex and age without signs of inflammation and the latter by subjects comparable for sex and age with evidence of systemic inflammation, marked by an increased plasma concentration of C-reactive protein (CRP).

## 2. Materials and Methods

### 2.1. Study Populations

**Figure 1:**
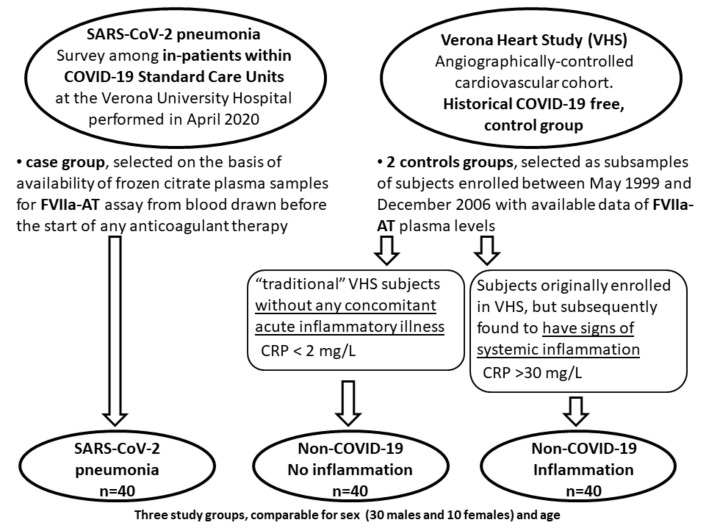
Schematic diagram of study design. Study design for the analysis of activated factor VII–antithrombin complex (FVIIa-AT) levels in patients with SARS-CoV-2 pneumonia.

The study design is summarized in Figure 1. We selected 40 patients (30 males and 10 females, mean age 60.6 ± 11.5 years) with SARS-CoV-2 pneumonia from a survey among in-patients within COVID-19 Standard Care Units at the Verona University Hospital, Italy, performed in April 2020. These subjects were selected on the basis of availability of frozen citrate plasma samples for FVIIa-AT assay from blood drawn before the start of any anticoagulant drugs, including thromboprophylaxis with low-molecular-weight heparin (LMWH). Subjects taking full-dose anticoagulant therapy (e.g., because of atrial fibrillation) were excluded from this study. Patients with SARS-CoV-2 pneumonia had no evident clinical manifestation of venous thromboembolism at time of enrollment, while during the stay within COVID-19 Standard Care Units, 8 patients (20%) were also diagnosed with deep vein thrombosis. Clinical information and laboratory data were collected at time of enrollment [10].

FVIIa-AT plasma levels, as well as other laboratory data of these COVID-19 patients, were compared with those of two different historical, COVID-19-free, control groups selected from Verona Heart Study (VHS) cardiovascular cohort as subsamples of subjects enrolled between May 1999 and December 2006, for whom data of FVIIa-AT plasma concentration were also available. The VHS is a regional survey that assessed new risk factors for coronary artery disease (CAD) in subjects with angiographic documentation of the state of their coronary vessels [25,26,29]. The subjects who were definitely enrolled in the VHS had no history of any acute illness, including acute coronary syndromes, in the month before the enrollment. They were clinically stable and had no signs of major systemic inflammation at laboratory assessment. However, in the screening phases some subjects were enrolled in VHS despite bearing concomitant inflammatory disorders. These subjects were detected after admission to VHS by evidencing increased levels of inflammatory biomarkers, namely CRP, and subsequent review of medical records (which mainly revealed upper airway infections), thereby being excluded from further analysis in VHS. 

In the context of the current analysis evaluating FVIIa-AT plasma levels in COVID-19, we selected from the former VHS population 40 CAD subjects, comparable for sex and age, without evidence of inflammation (CRP < 2 mg/L) and from the latter group 40 CAD subjects, comparable for sex and age, with signs of overt inflammation (CRP > 30 mg/L).

All VHS participants came from the same geographical area of northern Italy as the above-mentioned patients with COVID-19. At the time of blood sampling, a complete clinical history was collected, as well as data about drug therapies. Subjects taking any anticoagulant therapy were excluded from this study. 

### 2.2. Biochemical Analysis, FVIIa-AT and Coagulation Assays

In both COVID-19 and VHS population, samples of venous blood were drawn from each subject after an overnight fast, at the time of enrollment. Serum creatinine, CRP, and standard coagulation parameters, such as fibrinogen, PT, and aPTT were assessed with routine analytical methods, as specified in details elsewhere [26,30]. Glomerular filtration rate (GFR) was estimated from serum creatinine levels assessed with a standardized/compensated method by means of the four-variable version of the Modification of Diet in Renal Disease (MDRD) equation [31].

The concentration of FVIIa-AT was measured by ELISA (Asserachrom VIIa-AT; Diagnostica Stago, Asnieres, France) on frozen, never before thawed, citrate plasma samples for both COVID-19 and VHS populations. Venous blood samples were collected at the time of enrolment, processed within 1 h, stored in aliquots, and frozen at −80 °C. FVIIa-AT assay was performed on plasma samples previously thawed in a water bath at 37 °C for 5 min. All measurements were obtained in duplicate. The coefficients of variation, intra- and inter-assay, were <5%. Data of FVIIa-AT were available for all the subjects in the three cohorts included in this study. 

The activities of coagulation factor II (FII:c), factor V (FV:c), and factor VIII (FVIII:c) in COVID-19 patients were measured on an ACL TOP 750 analyzer (Instrumentation Laboratory, Werfen), while in non-COVID-19 subjects from VHS the same parameters were assayed with a similar method on a Behring Coagulation Timer (BCT, Dade Behring) by modification of the one-stage clotting method with the use of relative deficient plasma (Dade Behring). The intra- and inter-assay coefficients of variations were <5%. Results of factor activities were expressed in terms of IU/dL. Data of FII:c, FV:c, and FVIII:c were available for all the patients with SARS-CoV-2 pneumonia and for a subsample of subjects without COVID-19.

### 2.3. Statistical Analysis

All the calculations were performed using the IBM SPSS 23.0 (IBM Inc., Armonk, NY, USA) statistical package. Distributions of continuous variables in groups were expressed as mean ± standard deviations. Skewed variables, including FVIIa-AT, CRP, eGFR, PT, aPTT, and fibrinogen were logarithmically transformed, and then geometric means with 95% confidence intervals (CIs) were reported. Quantitative data among the three cohorts were assessed by Student’s *t*-test or by ANOVA, with Tukey’s post-hoc comparison of the means when indicated. Qualitative data were analyzed with χ^2^ test. Finally, a linear regression analysis with FVIIa-AT plasma concentration as dependent variable was performed in the whole study population to evaluate SARS-CoV-2 pneumonia/COVID-19 status as potential determinant of FVIIa-AT plasma levels. The strength of association between SARS-CoV-2 pneumonia/COVID-19 status and FVIIa-AT levels was assessed by calculating beta coefficients in linear regression models adjusted for potential confounders, i.e., sex, age, CRP, eGFR, and traditional coagulation parameters. A *p* value < 0.05 was considered significant.

## 3. Results

The study design is summarized in Figure 1. The main clinical and laboratory characteristics of the study populations are shown in Table 1.

**Figure 2:**
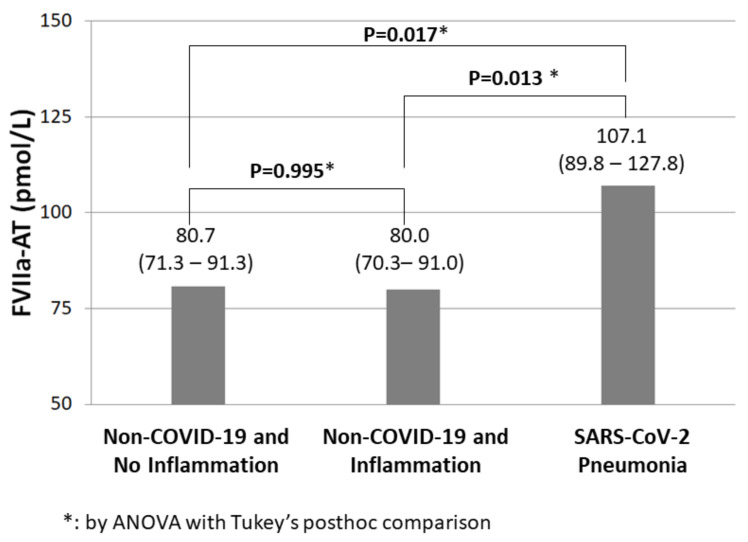
FVIIa-AT levels in subjects with or without SARS-CoV-2 infection. Activated factor VII–antithrombin complex (FVIIa-AT) plasma concentration in the three study groups.

There were no significant differences of gender, age, BMI, and eGFR among the three cohorts. Subjects with SARS-CoV-2 pneumonia had the highest levels of inflammatory biomarkers, namely CRP and fibrinogen (Table 1). D-dimer data were available only in subjects with SARS-CoV-2 pneumonia who, as expected, showed high D-dimer plasma levels (1400 with 95%CI 1128–1738 mcg/L). No correlation between D-dimer and FVIIa-AT levels was found in COVID-19 patients (R = 0.160, *p* = 0.343 by Pearson’s correlation test). Subjects with SARS-CoV-2 pneumonia had the highest plasma levels of FVIIa-AT, which were higher than those in subjects without COVID-19 either with or without inflammation (Figure 2). On the other hand, there was no difference in FVIIa-AT levels between non-COVID-19 subjects with or without inflammation (Figure 2). 

During the stay within COVID-19 Standard Care Units, eight patients (20%) were also diagnosed with deep vein thrombosis. There was no significant difference in FVIIa-AT plasma levels between subjects with or without a subsequent diagnosis of deep vein thrombosis (110.4 with 95%CI 67.1–182.0 versus 106.3 with 95%CI 87.1–129.7 pmol/L, *p* = 0.865 by Student’s *t*-test). Subjects with SARS-CoV-2 pneumonia had higher FVIIa-AT plasma levels as compared with those without COVID-19 either with or without inflammation also after exclusion of these eight patients with deep vein thrombosis (*p* = 0.022 and *p* = 0.028 by ANOVA with Tukey’s post-hoc comparison, respectively).

**Figure 3:**
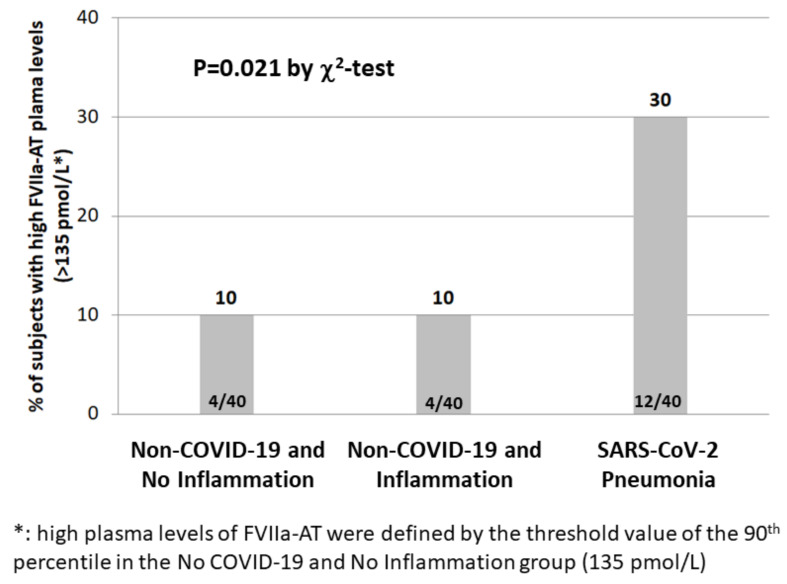
High FVIIa-AT levels in subjects with or without SARS-CoV2 infection. Prevalence of high-activated factor VII–antithrombin complex (FVIIa-AT) plasma levels in the three study groups.

Defining high levels of FVIIa-AT on the basis of the arbitrary threshold of the 90th percentile in subjects without COVID-19 and without signs of inflammation, subjects with SARS-CoV-2 pneumonia had a three-fold increased prevalence of high FVIIa-AT levels compared with non-COVID-19 subjects, either with or without inflammation (Figure 3). 

**Figure 4:**
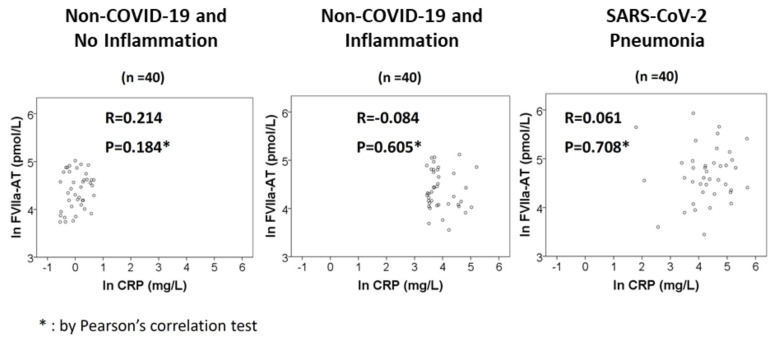
FVIIa-AT–CRP correlation. Correlations between activated factor VII–antithrombin complex (FVIIa-AT) and C-reactive protein (CRP) plasma levels in the three study groups.

No significant correlation was found between FVIIa-AT and CRP levels in any of the three study groups (Figure 4).

In subsample analysis (information on FII:c, FV:c, and FVIII:c was available only in a limited number of non-COVID-19 subjects), FII:c, FV:c, and FVIII:c were lower in subjects with SARS-CoV-2 pneumonia, with the highest FVIII:c levels in subjects without COVID-19 and with inflammation (Table 1). 

Considering the whole study population, SARS-CoV-2 pneumonia/COVID-19 status remained significantly associated with FVIIa-AT plasma levels by multiple linear regression models, even after adjustment for potential confounders, such as sex, age, CRP, eGFR, fibrinogen, PT, and aPTT (Table 2).

## 4. Discussion

COVID-19 is a multisystem pathology in which vascular endothelium is a critical target organ with predisposition to development of both micro- and macrothrombosis [19]. Coagulation abnormalities are hallmarks of COVID-19. Some coagulation biomarkers, such as D-dimer, are shown to have potential prognostic significance by predicting clinical outcomes and overall mortality in COVID-19 patients [32,33,34]. In this study, we showed that patients with SARS-CoV-2 pneumonia within Standard Care Units during the first pandemic wave had higher plasma levels of FVIIa-AT than historical, sex- and age-matched control groups (Figure 1 and Figure 2), thus supporting the hypothesis of an increased TF expression and TF-FVIIa interaction in COVID-19. 

As regards coagulation parameters, our data also include the assessment of FII:c, FV:c, and FVIII:c, which were lower in patients with SARS-CoV-2 pneumonia (Table 1), consistent with a condition of acute phase-related (presumably thrombotic) consumption of coagulation factors. However, the low number of subjects assessed for FII:c, FV:c, and FVIII:c in the control groups does not allow us to draw any firm conclusions. Therefore, although with significant limitations, the most robust result of our analysis should be considered the observed association of SARS-CoV-2 pneumonia with increased plasma levels of FVIIa-AT.

Our results are consistent with a few earlier reports on coagulation biomarkers in COVID-19. Francischetti and colleagues found a progressive increase in FVIIa-AT levels from controls to subjects with moderate and, lastly, severe SARS-CoV-2 infection [35]. Similarly, Willems and colleagues observed that FVIIa-AT plasma concentration remained elevated in about a third (35%) of recovered COVID-19 patients 3 months after SARS-CoV-2 infection [36].

Our data may support the concept of a crucial endothelial involvement in COVID-19 coagulopathy. Endothelial cells at rest do not constitutively express TF at their surface, though they can be fostered to express TF by several triggers, including inflammatory cytokines [37]. Endothelial cells are then a central joint of interaction between inflammation and coagulation, as well as being recognized as crucial players in the processes of immunothrombosis and thromboinflammation [38,39]. The intense inflammatory response triggered by SARS-CoV-2 can stimulate, activate, and damage vascular endothelium, as well as trigger coagulation pathways—including those linked with TF—by means of both endothelium-related and leukocyte-related mechanisms. As regards TF expression/activation, it is worthy of note that monocytes and macrophages also show very little or no basal/constitutive expression of TF, which can be induced by inflammatory stimuli [37]. 

Severe infections have been long known to cause haemostatic derangements, mainly by fostering systemic inflammation [40]. However, in our study population, the increase in FVIIa-AT in COVID-19 patients appeared to be independent of inflammatory markers, namely CRP (Table 2). In subjects with an elevated plasma concentration of CRP but without COVID-19, no increase in FVIIa-AT was observed (Figure 2 and Figure 3). Moreover, no correlation between FVIIa-AT and CRP levels was found in any of our cohorts (Figure 4). These results are consistent with previous data reported in the larger VHS cardiovascular cohort, in which no correlation between FVIIa-AT and CRP plasma levels was detected [25]. Notably, our results are also consistent with those reported by Willems and colleagues, who failed to find a significant correlation between coagulation biomarkers—such as FVIIa-AT—and inflammatory cytokines, thus suggesting a kind of dissociation between thrombotic and inflammatory states in COVID-19 [36]. It is hence tempting to speculate that SARS-CoV-2 infection may directly or indirectly stimulate TF expression by endothelial cells and/or leukocytes independent of its associated cytokine storm in patients with severe illness [36]. Coronaviruses may cause angiotensin-converting enzyme 2 down-regulation, resulting in increased levels of angiotensin 2 [41], which in turn can induce TF overexpression [42]. Moreover, the activation of toll-like receptor 3 in the presence of double-stranded RNA viruses can induce TF expression in cultured endothelial cells, thus inducing a procoagulant state in the endothelium [43]. All these molecular mechanisms represent potential biological grounds supporting the hypothesis of TF upregulation, even beyond classic inflammatory pathways, in the multifactorial COVID-19 coagulopathy. Notably, the results of high FVIIa-AT plasma levels in COVID-19 patients were confirmed even after the exclusion of subjects with a subsequent diagnosis of deep vein thrombosis, thereby allowing us to limit the potential biases related to acute thrombotic events.

Being as FVIIa-AT is an indirect marker of FVIIa-TF interaction, its laboratory evaluation could hence be considered clinically useful in conditions such as COVID-19 characterized by TF upregulation/activation, and where the inhibition of the TF pathway has been hypothesized to exert beneficial effects [44]. It is worthy of note that increased plasma FVIIa-AT levels have been associated with the increased risk of mortality in different clinical settings, thus proving to be a potential prognostic biomarker of high-risk conditions [25,27]. In 2016, observing the increased risk of total and cardiovascular mortality in VHS subjects with clinically stable CAD and high FVIIa-AT plasma levels, we hypothesized a possible beneficial use of FXa inhibitors in CAD patients, supported by blocking the excess of FX activation mediated by TF–FVIIa interaction, which is indirectly marked by high FVIIa-AT plasma levels. In 2017, the COMPASS trial demonstrated that low-dose rivaroxaban, a FXa inhibitor, can improve clinical outcomes in patients with stable atherosclerotic vascular disease [45]. Low-molecular-weight heparins, which are characterized by predominant anti-FXa activity, are already recognized as a cornerstone in the management of COVID-19, although many questions remain about the optimum approach, dose, and duration of therapy [46]. According to all the previous considerations, we are tempted here to speculate that subjects with SARS-CoV-2 pneumonia and high FVIIa-AT plasma levels may have the greatest benefit from treatments targeting FXa inhibition.

Our study has several limitations that should be acknowledged, the first of which is the small sample size and the limited number of assessed coagulation biomarkers. The differences in FVIIa-AT plasma levels among the groups, although statistically significant, showed a substantial overlap of values. Moreover, the COVID-19 cohort was represented by patients with SARS-CoV-2 pneumonia, while the control group without COVID-19 and with inflammation included mainly subjects with upper respiratory tract infections, without pneumonia. Therefore, we cannot exclude that the alterations in FVIIa-AT levels may be linked to the general condition of pneumonia, rather than being specific to SARS-CoV-2 infection. A control group of patients with pneumonia not due to SARS-CoV-2 infection would be ideal for such comparison. Nonetheless, our results are consistent with those of other studies showing increased FVIIa-AT levels in and after SARS-CoV-2 infection [35,36], thereby supporting the hypothesis of an increased TF-FVIIa interaction in COVID-19. Moreover, according with earlier reports showing a dissociation between coagulation and inflammation parameters [36], our data suggest also that the activation of the TF pathway in COVID-19 may be, at least in part, independent of inflammatory mechanisms. 

In summary, by showing increased plasma levels of FVIIa-AT in patients with SARS-CoV-2 pneumonia, our data address the possible role of the TF pathway in COVID-19. Our results need to be validated by further larger studies, with a prospective design evaluating the potential prognostic significance of FVIIa-AT plasma levels in subjects with SARS-CoV-2 infection and also comparing coagulation biomarkers among the different COVID-19 pandemic waves, which are known to be characterized by different clinical phenotypes and outcomes in the current scenario. If confirmed, the results of the present study may pave the way for further investigations and contribute to advancing the understanding of the multifaceted pathophysiology of COVID-19.

## Figures and Tables

**Table 1:** Study cohort characteristics. Clinical and laboratory characteristics of the three study groups: (i) non-COVID-19 subjects without inflammation, (ii) non-COVID-19 subjects with inflammation, and (iii) subjects with SARS-CoV-2 pneumonia.

	Non-COVID-19 No Inflammation (n = 40)	Non-COVID-19 Inflammation (n = 40)	SARS-CoV-2 Pneumonia (n = 40)	*p* *
Female sex (%)	25.0	25.0	25.0	NS
Age (years)	60.6 ± 11.5	60.9 ± 12.1	64.8 ± 12.3	NS
BMI (kg/m^2^)	26.5 ± 3.5	27.0 ± 3.9	26.8 ± 4.4	NS
CRP (mg/L)	0.9 (0.7–1.2)	51.6 (44.0–60.4)	71.5 (54.4–93.8)	<0.001
eGFR (mL/min) #	73.9 (67.9–80.4)	71.8 (60.9–80.6)	70.8 (63.6–78.8)	NS
PT	0.98 (0.97–1.00)	0.97 (0.95–1.00)	1.10 (1.07–1.14)	<0.001
aPTT	0.97 (0.94–1.01)	1.03 (0.98–1.08)	1.01 (0.97–1.06)	NS
Fibrinogen (g/L)	3.16 (2.95–3.37)	5.33 (4.67–6.07)	6.19 (5.48–7.00)	<0.001
FVIIa-AT (pmol/L)	80.7 (71.3–91.3)	80.0 (70.3–91.0)	107.1 (89.8–127.8)	0.006
FII:c ^	120.0 ± 24.5	118.4 ± 24.2	82.9 ± 19.7	<0.001
FV:c ^	140.7 ± 52.2	142.2 ± 25.3	99.8 ± 23.3	<0.001
FVIII:c ^	148.9 ± 60.7	200.9 ± 66.6	65.2 ± 21.9	<0.001

CRP: C-reactive protein; eGFR: estimated Glomerular Filtration Rate; PT: Prothrombin Time; aPTT: activated Partial Thromboplastin Time; FVIIa-AT: activated factor VII-antithrombin complex; FII:c: factor II coagulant activity; FV:c: factor V coagulant activity; FVIII:c: factor VIII coagulant activity; NS: not significant. *: by ANOVA or χ2-test, when indicated. #: eGFR was calculated by means of Modification of Diet in Renal Disease (MDRD) equation. ^: Data of FII:c, FV:c, and FVIII:c were available for all the 40 subjects with SARS-CoV-2 pneumonia, 25 subjects within non-COVID-19 and no inflammation group, and in 9 subjects within non-COVID-19 and inflammation group.

**Table 2:** SARS-CoV-2 infection as determinant of FVIIa-AT plasma levels. Strength of association between SARS-CoV-2 pneumonia and FVIIa-AT plasma levels in the whole study population by linear regression models.

SARS-CoV-2 Pneumonia as Determinant of FVIIa-AT Levels by Linear Regression Models		Beta Coefficient	95% CI	*p*
Unadjusted		0.288	0.114–0.461	0.001
Adjusted	Model 1	0.276	0.100–0.452	0.002
	Model 2	0.265	0.056–0.474	0.014
	Model 3	0.264	0.055–0.473	0.014
	Model 4	0.322	0.055–0.589	0.019

Model 1: adjusted for sex, age. Model 2: adjusted for sex, age, CRP. Model 3: adjusted for sex, age, CRP, eGFR. Model 4: adjusted for sex, age, CRP, eGFR, fibrinogen, PT, aPTT. CI: Confidence Interval, CRP: C-Reactive Protein, eGFR: estimated Glomerular Filtration Rate, PT: Prothrombin Time, aPTT: activated Partial Thromboplastin Time.

## Data Availability

The data presented in this study are available on request from the corresponding author. The data are not publicly available due to privacy reasons.
